# Great Desire for Extended Life and Health amongst the American Public

**DOI:** 10.3389/fgene.2015.00353

**Published:** 2016-01-20

**Authors:** Yoni Donner, Kristen Fortney, Stuart R. G. Calimport, Karl Pfleger, Munjal Shah, Joe Betts-LaCroix

**Affiliations:** ^1^Department of Computer Science, Stanford UniversityStanford, CA, USA; ^2^Department of Developmental Biology, Stanford UniversityStanford, CA, USA; ^3^School of Life and Health Sciences, Aston UniversityBirmingham, UK; ^4^Independent ResearcherSan Francisco, CA, USA; ^5^Health Extension FoundationSeattle, WA, USA

**Keywords:** survey research, life extension, public policy, public opinion, lifespan

## Abstract

People want to live long, healthy lives. Previous surveys suggest very limited interest in much longer lifespans, but we show that stipulating good health changes responses to favor longer lives by an order of magnitude. Advances in aging research hold out hope for greatly slowed aging with associated good health. Understanding the public's desires correctly is important to avoid misallocation of resources for research.

Recent advances in aging research and regenerative medicine may soon translate into dramatically increased human lifespans. But does the American public want to live longer? Popular press argues the answer is no, e.g., a recent survey on desired lifespan reported in the New York Times found 60% of respondents voted for the shortest option, an 80 year lifespan, while fewer than 1% opted for an unlimited lifespan (Duncan, 2012). Here, we show that negative attitudes to longer lives are a consequence of erroneously equating extended life with an extended period of frailty. When we stipulated continued health to the original survey question, responses dramatically favored longer life: only 20% wish to die at age 85, while 42% want an unlimited lifespan. Since funding for aging research depends on its perceived value, better science communication is needed to align public policy with public interests.

We surveyed 1000 individuals (through “Ask Your Target Market,” http://aytm.com/) about how long they wished to live (to age 85, 120, 150, or indefinitely), under 3 scenarios: (1) sustained mental and physical youthfulness, (2) mental youthfulness only, (3) physical youthfulness only. While responses to the two partial youthfulness conditions recapitulated the results of previous surveys (Cicirelli, 2011; Kogan et al., 2011; Partridge et al., 2011; Duncan, 2012; Pew Research Center, 2013), i.e., most responders (65.3%) wished to live to age 85 only—under scenario (1) the pattern of responses was completely different. When guaranteed mental and physical health, 797 of 1000 people wanted to live to 120 or longer, and 53.1% of the 797 desired unlimited life spans. Furthermore, 70.1% of the people who responded 85 to scenario (2) or (3) changed their answer to 120 or longer in scenario (1). Full survey response data are publicly available from: http://healthextension.co/wp-content/uploads/2015/11/AYTM-Results.csv.

The fraction of people who changed their answer from 85 to 120 or longer was significantly higher among people with some interest in science (445/622 vs. 13/31, *p* < 0.001, Fisher's exact test), and this was the main predictor of changing the answer to favor longer life. Less significant correlations were found with other surveyed variables such as age, health status, and self-esteem. Similar results were recently reported for Canadians (Dragojlovic, 2013): 59% of 1231 respondents wished to live to 120 (the maximum age included in that survey), and science orientation was the strongest predictor of support for life extension.

We also reproduced our primary finding—that most people wish to live far longer than the average human lifespan so long as they stay healthy—using Google Surveys (McDonald et al., 2012). In this replication cohort of 1500 respondents, we found that 74.4% wished to live to 120 or longer if health was guaranteed, but only 57.4% wished to live that long if it wasn't. Full survey data and results are publicly available in an interactive browsable format from: https://www.google.com/insights/consumersurveys/view?survey=rkiemlpdkjgfe.

A recent survey by the Pew foundation (Pew Research Center, 2013) found a basic result similar to other prior work: 56% of people would not want medical treatments to slow aging vs. 38% who would. Interestingly, this survey also asked what respondents thought other people would do, finding that 68% thought most others would choose such treatments (vs. 27% who did not). Future work to explore this discrepancy should investigate whether most people perceive a difference in likely future health of others vs. themselves in the context of longer lives. The Pew survey also found that more people thought radical slowing of aging would be bad for society (51%) than good (41%). Future work should test whether this too would reverse under stipulation of youthful health.

Results from The Human Memome Project (HMP)—a project to survey longevity predictors, socio-cultural information and attitudes to longevity—show that attitudes to long life are not just positive in North America, but that globally, citizens show positive attitudes to long life (Calimport and Bentley, 2013). 175/394 participants from crowdsourcing and citizen science communities definitely wanting to live as long as possible and 97/394 participants probably wanting to live as long as possible. 189/394 participants stated that they definitely valued their lifespan and that of others highlighting the value of lifespan for citizens. The HMP data for those that opted in to the open science dataset are available on ResearchGate at: https://www.researchgate.net/publication/256460492_HMP_Open_Science_Dataset_04092013.

The public wants to live long, and live healthy. Human supercentenarians give some of the best evidence for the possibility of increased healthspan and healthy aging, or compression of morbidity (Fries, 1980). For example, looking at all age-related diseases in aggregate, Andersen et al. (2012) found that supercentenarians suffered from these only for the last ~5% of their lives, while controls were sick for ~18%. Making healthy aging a reality for the rest of the population will be scientifically challenging. Nevertheless, it is becoming increasingly more necessary: chronic age-related diseases account for 75% of Medicare spending, and these numbers are projected to rise as baby boomers age [10]. The NIA currently receives less than 1% of the NIH's overall annual budget, or less than 0.05% of annual Medicare spending; this is a misallocation of resources (Stipp, 2012). There is a growing demand for more awareness and more funding for basic aging research, and new initiatives such as the Healthspan Campaign (http://healthspancampaign.org/) and the trans-NIH Geroscience Interest Group (http://sigs.nih.gov/geroscience/) are helping lead the way forward. Investing in scientific research and development that targets aging, the process underlying multiple chronic diseases, can offer uniquely high potential returns.

## Author contributions

Conceived and designed the experiments: JB, MS, KP, SC. Analyzed the data: YD, KF. Wrote the paper: YD, KF, SC, KP, MS, JB.

### Conflict of interest statement

The authors declare that the research was conducted in the absence of any commercial or financial relationships that could be construed as a potential conflict of interest.
